# Postoperative Gastric Perforation in a Newborn with Duodenal Atresia

**DOI:** 10.21699/jns.v5i4.469

**Published:** 2016-10-10

**Authors:** Anko Antabak, Marko Bogović, Jurica Vuković, Ruža Grizelj, Vinka Barbarić Babić, Dino Papeš, Tomislav Luetić

**Affiliations:** 1 Department of Surgery, Clinical Hospital Center Zagreb, Zagreb, Croatia; 2 Department of Pediatrics, Clinical Hospital Center Zagreb, Zagreb, Croatia; 3 Department of Radiology, Clinical Hospital Center Zagreb, Zagreb, Croatia

**Keywords:** Gastric perforation, Neonate, Duodenal atresia

## Abstract

Gastric perforation (GP) in neonates is a rare entity with high mortality. Although the etiology is not completely understood, it mostly occurs in premature neonates on assisted ventilation. Combination of duodenal atresia and gastric perforation is very rare. We present a case duodenal atresia who developed gastric perforation after operetion for duodenal atresia. Analysis of the patient medical record and histology report did not reveal the etiology of the perforation.

## CASE REPORT

A one-day-old full-term male newborn, weighing 3400g, was admitted with suspicion of duodenal atresia due to history of polyhydramnios and antenatal ultrasound findings. An 8Fr nasogastric (NG) tube was inserted and prophylactic antibiotic (ceftriaxone) was initiated. A supine abdominal radiograph and contrast study revealed a double-bubble sign (Fig.1A). At laparotomy, the atresia was detected in the pre-ampullary region of the duodenum, and diamond-shaped duodeno-duodenostomy was performed. NG tube was passed across the anastomosis. Abdominal distension, tachypnea and respiratory distress occurred suddenly 24 hours after surgery. Abdominal radiography showed massive pneumoperitoneum. Emergent re-surgery found a linear tear (5 cm) along greater curvature of the posterior gastric wall (Fig.2A). The edges of the tear seemed very thin, as the muscular layer was missing. Perforation edges were excised until normal gastric wall was reached (approximately 1 cm from edge) and primary repair of the perforation was performed in two-layer (Fig.2B). Further postoperative course was uneventful. Upper GI series performed after seven days (Fig.1B) was normal so NG tube was removed, breastfeeding was started, and he was discharged on the 14th postoperative day. Histological analysis of the excised perforation edges showed gastric wall necrosis. 

**Figure:**
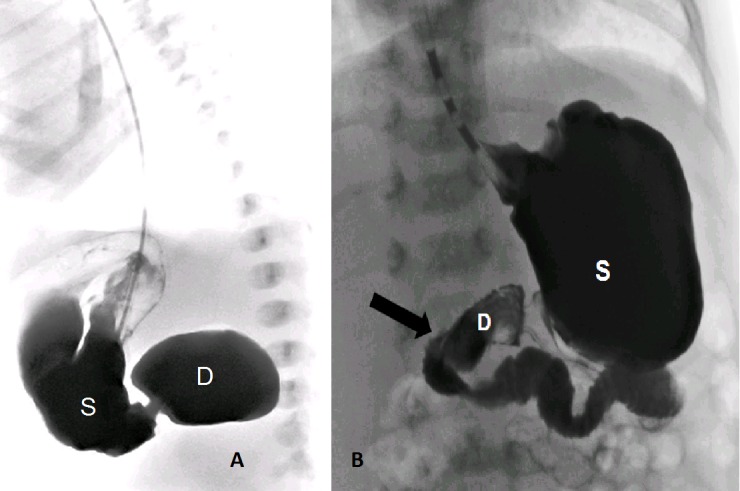
Figure 1: A) Contrast study showing duodenal atresia. B) Postoperative contrast study showing intact stomach and duodenal repairs.

**Figure:**
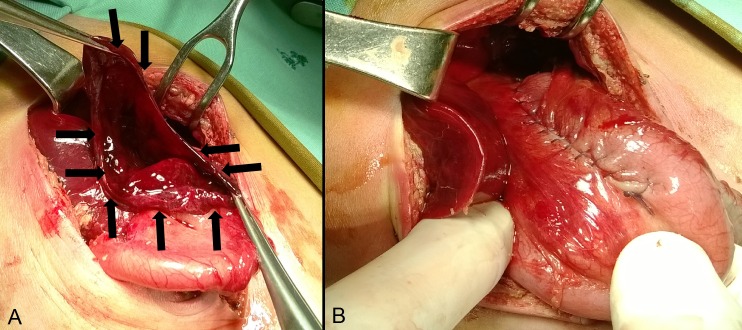
Figure 2: A) intraoperative view of the stomach rupture, B) after repair

## DISCUSSION

Neonatal GP is a rare entity which requires prompt diagnosis and surgical intervention. The exact etiology of the condition is unclear, although various theories have been suggested including congenital absence of the muscular layer of stomach, increased intraluminal pressure due to distal obstruction by atresia, stenosis or bands, positive pressure mask ventilation during resuscitation, all have been proposed. Vigorous NG tube placement occasionally causes GP, which usually appears as a puncture wound or as a short tear. Prematurity and perinatal stresses are the most important risk factors for GP because of a possible low-perfusion state that leads to gastric wall injury. However, predisposing factors can not be identified in around 20% of the patients [1-8]

Congenital duodenal atresia complicated by a GP is a very rare condition. According to a case report and literature review by Takebayashi, there were 12 reported cases of GP related to duodenal atresia, between 1940 and 1975, and one additional case was reported afterwards [9,10]. Only three of the reported 13 cases survived. In our patient, although the stomach had a wide perforation, generalized peritonitis was absent. Preoperative antibiotic use, early diagnosis of the GP and the pre-ampullary type of duodenal atresia might have prevented peritoneal irritation by pancreatic and bilious fluids. Our patient had two risk factors for the neonatal GP: duodenal obstruction and NG tube insertion. However, we do not believe that any of those had caused GP in our patient. The NG tube had a proper length and was placed under direct vision during the first surgery. In addition, the perforation type was different than NG tube related perforation, which generally has a small diameter. Increased intra-gastric pressure due to distal obstruction too cannot stand as an etiology as the stomach was decompressed by NG tube.

To conclude, neonatal GP may occur after successful treatment of duodenal atresia without clear etiology. Revision surgery performed as soon as possible and intensive treatment is crucial for good outcome.

## Footnotes

**Source of Support:** Nil

**Conflict of Interest:** Nil
